# Moral disagreement and the limits of AI value alignment: *a dual challenge of epistemic justification and political legitimacy*

**DOI:** 10.1007/s00146-025-02427-2

**Published:** 2025-06-21

**Authors:** Nick Schuster, Daniel Kilov

**Affiliations:** Australian National University, Canberra, Australia

**Keywords:** Artificial intelligence, Moral disagreement, Moral epistemology, Political legitimacy, Value alignment

## Abstract

AI systems are increasingly in a position to have deep and systemic impacts on human wellbeing. Projects in value alignment, a critical area of AI safety research, must ultimately aim to ensure that all those who stand to be affected by such systems have good reason to accept their outputs. This is especially challenging where AI systems are involved in making morally controversial decisions. In this paper, we consider three current approaches to value alignment: crowdsourcing, reinforcement learning from human feedback, and constitutional AI. We argue that all three fail to accommodate reasonable moral disagreement, since they provide neither good epistemic reasons nor good political reasons for accepting AI systems’ morally controversial outputs. Since these appear to be the most promising approaches to value alignment currently on offer, we conclude that accommodating reasonable moral disagreement remains an open problem for AI safety, and we offer guidance for future research.

## Introduction

When artificial intelligence (AI) systems are in a position to profoundly impact human wellbeing, the safety of such systems requires that they be not only technically robust—that is, reliable under adverse conditions—but also appropriately aligned with human values. An AI surveillance system, for example, may be thoroughly reliable yet deeply dangerous if used by an authoritarian government to suppress political dissidents. Such systems are safe only if they are acceptable from the point of view of the surveilled as well as the surveillants. In such cases, then AI value alignment must aim to ensure that all who stand to be impacted by AI have good reason to accept its outputs. And as AI becomes more involved in impactful decision-making, especially about morally controversial matters, value alignment is an increasingly critical as well as increasingly challenging aspect of AI safety.

In this paper, we focus on three prominent approaches to AI value alignment: crowdsourcing, reinforcement learning from human feedback (RLHF), and constitutional AI. These methods promise to imbue AI with good moral judgment, and they can have the guise of democratic processes. Yet, we argue, all three ultimately fail to accommodate reasonable moral disagreement. Despite appearances, the outputs of AI systems aligned via these approaches are neither epistemically justified nor politically legitimate, and so those who reasonably disagree with them lack good reason to accept them.

We begin our critique by explicating the challenge for value alignment when AI systems’ outputs are systemically impactful as well as morally controversial. And we specify two kinds of reasons people can have for accepting such outputs: moral-epistemic reasons and political reasons. We then consider whether the crowdsourcing approach can provide either kind of reason. We argue that, though crowdsourcing has epistemic value for certain AI applications, this does not extend to making morally controversial judgments and decisions; and though it allows the general public to provide input, this is not enough to legitimize AI’s systemically impactful outputs. We then argue that the same problems apply to both RLHF and constitutional AI. Since these appear to be the most promising approaches to value alignment currently on offer, we conclude that accommodating reasonable moral disagreement remains an open problem for AI safety. And we close by offering guidance for future research.

## A challenge for AI value alignment

We understand AI to be *systemically impactful* insofar as it not only stands to have significant impacts on human wellbeing but can also embed systemic biases into social systems and institutions by distributing its impacts in potentially unjust ways. And we understand *reasonable moral disagreement* to be grounded in opposing moral worldviews which are, nonetheless, both internally coherent and compatible with basic liberal values, like freedom of thought, respect for individual rights, and tolerance of diverse conceptions of the good. We follow John Rawls (2001) in taking this sort of “reasonable pluralism to be a permanent condition” of diverse, modern societies (p. 33). The challenge for value alignment we wish to draw attention to, then, is how to accommodate reasonable moral disagreement when AI systems are involved in making controversial judgments and decisions that have systemic impacts across pluralistic societies.^1^

AI systems are already being used to carry out military operations, allocate medical resources, drive cars, sentence criminal suspects, make hiring and admissions decisions, process loan applications, distribute social services and benefits, and perform many other tasks that profoundly affect people’s lives and life prospects.^2^ Much of the appeal of using AI for such applications is its efficiency and scalability. Self-driving cars, for instance, could deliver the benefits of taxi services at a fraction of the cost. What is more, AI systems promise to drive more safely than humans. And even when accidents are unavoidable, they could make more fine-grained and systematic decisions about whom to endanger than humans ever could (Awad et al. 2018). The same goes for unavoidable tradeoffs in medical resource allocation, military combat, sentencing, hiring, and so forth.

This exciting prospect is due to AI’s superhuman capacities: it can rapidly process a vast amount of information, systematically act according to highly complex decision procedures, and be implemented at scale. We humans are much more limited in these respects. We evidently tend to overlook morally relevant information and fail to respond systematically to moral factors even in relatively straightforward situations.^3^ And even if each of us were more reliable in these ways, people with different moral views would surely judge and act differently from each other in many cases.

Such limitations make human decision-making problematically inconsistent. Whether someone gets a traffic ticket or a mere warning, a passing or failing grade, even a new kidney, can depend largely on who makes the decision and under what extraneous conditions; whereas, AI can apply the same fine-grained decision procedures systematically across myriad situations. AI could, therefore, transform many areas of human life for the better (Sinnott-Armstrong and Skorburg 2021). For example, human factors in car-based transportation impose risk with substantial randomness: who or what gets destroyed in any given accident cannot be precisely predicted or controlled. Traditional safety measures, therefore, focus on preventing accidents in the first place and limiting harm when they occur, but they stop short of distributing unavoidable risk. AI makes this further safety measure a practical possibility. And supposing that some distributions of risk are better than others, choosing *not* to take such measures would be plainly irresponsible. If self-driving cars can be made to protect people over property, for instance, they should be.

But suppose they were also made to protect their passengers at the expense of pedestrians. Of course, human drivers sometimes do the same. The problem AI introduces here is that the risk pedestrians face would become less random and more *systemic—*that is, embedded in the transportation system rather than incidental to it—as self-driving cars become increasingly prevalent on public roads. In the same way, AI systems for military, medical, criminal justice, financial, and many other applications stand to systemically favor some groups over others where people would otherwise be subject to more random outcomes (Eubanks 2018).

So, AI’s ability to make extremely fine-grained yet systematic decisions cuts both ways. It could make things either much better or much worse, depending on whether AI systems are appropriately aligned with human values. This is why value alignment is so critical for AI safety. The reason it is so challenging is that people can and do disagree about how AI systems should behave. For example, people evidently disagree about whether self-driving cars should protect younger people at the expense of older people.^4^ And opposing positions here, while at odds with each other, are nonetheless both reasonable. That is, they are both grounded in coherent moral worldviews which, despite their points of disagreement, both warrant tolerance and respect in a pluralistic liberal society.^5^

If AI is used to make fine-grained, morally controversial decisions at scale in such a society, then, many will reasonably judge it to be systemically wrong, whatever it does. It must take sides, one way or another, on myriad contentious issues. This challenge for AI value alignment can therefore be understood as an instance of the more general political challenge of accommodating reasonable moral disagreement as diverse societies decide how to live together (Gabriel 2020). When AI operates at a scale that amounts to a form of governance, it becomes subject to norms of governance. In addition to being “safe” in the narrow sense of “technically robust,” then, systemically impactful AI must also satisfy standards of public justification and legitimacy (Gabriel & Ghazavi 2022). To the extent that it fails to do so, it poses an authoritarian threat.

At the same time, though, the moral promise of AI lies in its ability to handle particular cases far more efficiently and in far more fine-grained detail than cumbersome political processes like voting; and its scalability enables it to systematically handle problems that traditional public institutions never could. Transportation and police departments, for example, can implement and enforce traffic laws, but they cannot control every movement of every vehicle on the road. AI practically could. And it could exercise similarly fine-grained yet scalable control over many other matters that previously eluded this level of governance. Again, it would be hard to justify *not* leveraging the power of AI to distribute the costs, benefits, risks, and opportunities of living in civil society better than humans and human institutions can, *as long as all who stand to be affected by it have good reason to accept its outputs*.

If current projects in value alignment meet this challenge, they could usher in an unprecedented era of justice and wellbeing facilitated by AI technocracy. They could “embed the general will into an algorithmic social contract” (Rahwan 2018, p. 8), enabling us as never before “to decide, as a community, what we believe to be right or wrong” (Awad et al. 2018, p. 63). But if they fail, AI is apt to become an increasingly authoritarian centralizing force, locking unacceptable values into both public and private institutions which together govern nearly every aspect of human life (Hendrycks et al., pp. 9–10). So, how could AI’s systemically impactful judgements and decisions be made acceptable, even to those who reasonably disagree with them? There are two kinds of reasons people can accept such outputs: moral-epistemic reasons and political reasons.^6^

Epistemically, if we have good reason to think that the judgments and decisions of an AI system are likely to be *morally correct*, then we have good reason to accept them. This is the same kind of reason we have for accepting the judgments and decisions of human experts, like airline pilots and surgeons, even when they contradict our own inexpert views. As long as we have good reason to believe that such experts know better than we do, we have good epistemic reason to defer to them. Likewise, if we have good reason to think that an AI system “knows” better than we do regarding moral matters, then we have good moral-epistemic reason to defer to it as a moral authority.

Alternatively, if we have good reason to think that the outputs of an AI system are *democratically legitimate*, then we have good *political* reason to accept them. This is the same kind of reason we have for accepting the outcomes of elections and other democratic procedures, even when our preferred candidate or policy does not prevail. We do not have to think that such outcomes are morally correct, then, to accept them for political reasons. As long as we can reach a reasonable overlapping consensus about how to make such decisions together, we have reason to accept them on political grounds alone (Rawls 2001). Likewise, if we could agree on fair procedures for AI value alignment, this could give us good political reason to accept the outputs of AI systems even when we think they are morally wrong.

Thus, moral-epistemic reasons and political reasons could each make morally controversial outputs of AI systems generally acceptable. Like the authoritative expertise of an airline pilot, a well-aligned AI system’s moral expertise may warrant our deference. Or alternatively, like an election, the value alignment process could legitimize the system’s outputs, giving us political reason to accept them. And importantly, for the purposes of this paper, we allow that good reason of either kind for accepting AI systems’ outputs is sufficient to vindicate a given approach to value alignment. It need not satisfy the higher standard of giving people reason they *cannot reject*. We now consider whether current approaches can satisfy this lower standard.

## Crowdsourcing

The crowdsourcing approach to value alignment is inspired by the idea that AI systems could be optimized for moral judgment and decision-making via the same methods used to train them to make other kinds of judgments and decisions at expert, even superhuman, levels. If those who stand to be affected by AI systems had good reason to think they were indeed moral experts, then they could accept their outputs on moral-epistemic grounds. More precisely, if people had good reason to believe that an AI system’s judgments and decisions were sufficiently likely to be *morally correct*, then they would have good reason to accept them even when they disagreed with them.^7^

Such a system would be analogous to many others currently in use, such as those used to diagnose melanomas: insofar as people have reason to think these expert systems are likely to be correct, they have reason to accept their outputs as authoritative, regardless of their own comparatively inexpert judgments. In this section, we first discuss a method for training such expert systems which relies on identifiable human experts, and we explain why moral expertise, in particular, poses a challenge here. We then outline the crowdsourcing approach and explain how it purports to avoid this challenge.

### Automating moral expertise: a challenge

MedEthEx is an instructive early attempt to automate moral expertise with AI. A professional medical ethicist trains MedEthEx by responding to various scenarios a physician might encounter, such as a patient refusing treatment. For each scenario, the trainer evaluates possible responses by assigning intensities to each of four moral duties—respect for autonomy, non-maleficence, beneficence, and justice—and indicates which response she judges to be right, all things considered.^8^ MedEthEx then uses this training data to update a decision procedure, or algorithm, that explains all of its trainer’s judgments in terms of the intensities she assigns to the relevant duties, via inductive logic programming, an early form of machine learning (ML). Ultimately, the goal is to refine an algorithm that can provide sound moral guidance in a range of novel cases by predicting what the trainer would advise (Anderson et al. 2006).

One notable limitation of MedEthEx, however, is that it requires a definitive response for each training case, and so it cannot learn from two trainers who disagree with each other. What MedEthEx aims to provide, then, is just an approximation of what the particular medical ethicist who trained it would advise in novel cases. But medical ethicists can and do disagree with each other, not just about specific cases but general principles too, and even overarching ethical frameworks (MedEthEx’s four duties are themselves contestable). MedEthEx is not designed to adjudicate such disagreements. What to do, then, when purported moral experts disagree?

One could try to determine who the real moral experts are and allow AI to learn only from them. The nature of moral expertise, however, is heavily debated.^9^ In fact, some doubt that it exists at all.^10^ And even if these debates were settled in favor of some particular conception of moral expertise, it still would not be clear how to distinguish genuine moral experts from their inferiors (Cholbi 2007). Suppose, for instance, that Peter Singer (1972) is right that moral philosophers have a special claim to moral expertise, because:

Someone familiar with moral concepts and with moral arguments, who has ample time to gather information and think about it, may reasonably be expected to reach a soundly based conclusion more often than someone who is unfamiliar with moral concepts and moral arguments and has little time. (p. 117)

Even so, philosophers widely disagree with each other, not just about first-order moral judgments, but higher order moral principles too, and even the very nature of morality (Bourget and Chalmers 2023). Such disagreement is also readily apparent among other plausible candidates for moral expertise: religious leaders, social critics, and policy makers, for example.

What is more, moral disagreements often cannot be resolved by the standards typically appealed to in other domains of expertise, such as independent verification and expert consensus (McGrath 2008). If two dermatologists, for instance, disagree about whether a mole is cancerous, it can be biopsied. And if they disagree about the best course of treatment, this might be settled by appealing to standards of care widely endorsed by their profession. But because resolving disagreements among purported moral experts is not as straightforward as this, distinguishing correct attributions of moral expertise from specious ones *ex ante* looks to be a non-starter. It would be better, then, if AI could be optimized for moral judgment and decision-making without needing to be trained by moral experts specifically. The crowdsourcing approach to value alignment promises to do just that, drawing on the wisdom of the crowd to render expert moral judgments and decisions, even where humans (including purported moral experts) reasonably disagree.

### The wisdom of the crowd

Advanced machine learning techniques, like deep learning with artificial neural networks, enable AI to discover highly complex patterns in massive datasets, which it can then exploit for a wide range of purposes. For example, AI voice and image recognition systems train on thousands of audio clips and images to find patterns which enable them to accurately determine what people say or what images contain in novel cases. These training data are standardly labeled via crowdsourcing, a process whereby many human crowdworkers make judgments about their contents and label them accordingly. AI systems then learn how to match audio clips and images to labels correctly. The simplest method for this is supervised learning, where the system guesses, more-or-less randomly at first, and adjusts its algorithm after each attempt according to whether its guess matches the relevant label.

For our purposes, we highlight two key advantages of this approach over the one employed by MedEthEx. First, crowdworkers need not have any particular expertise to train AI systems to perform at expert, even superhuman, levels. And second, the training data can contain cases about which crowdworkers disagree, yet this does not prevent AI systems from learning to reliably get things right in such cases. For example, it is hard for humans to recognize blurry faces (hence the widespread use of blurring faces for anonymity). So crowdworkers are apt to disagree about whose face a blurry image contains, and the labels they attach to such images can reflect this disagreement. But AI systems can easily identify blurry faces correctly once they have undergone enough supervised learning with clearer images of the same people, where crowdworkers can reliably attach the right labels (McPherson et al. 2016).

To explain AI’s ability to outperform humans by learning from the wisdom of the crowd in this way, ML researchers have appealed to the Condorcet jury theorem:

If each voter has a probability p of being correct and the probability of a majority of voters being correct is P, then p>0.5 implies P>p. In the limit, P approaches 1, for all p>0.5, as the number of voters approaches infinity (Cunningham et al. 2008).

In plain terms, we could say that as long as crowdworkers are at least minimally competent at labeling data for supervised learning (that is, they get the labels right more often than not), the resultant AI will be more competent than the typical crowdworker. And the more training data they provide, the better it will be. In the facial recognition case, crowdworkers just need to label enough clear images correctly for AI to discover patterns among them which extend to blurry images.

In principle, the same reasoning applies to AI systems trained on crowdsourced moral judgments. As long as crowdworkers can make good moral judgments about enough clear, uncontroversial cases, the resultant AI should be better than the typical crowdworker at making good moral judgements, even about harder, more controversial cases. And the more training data of this kind the crowdworkers provide, the better it should be at moral judgment. This, then, is how AI might achieve moral expertise even without input from human moral experts. For illustration, consider two prototypical systems that use crowdsourcing for value alignment: Delphi and the Moral Machine.

Delphi generates moral judgments in response to natural language queries. The user types a prompt like “eating meat” or “lying to a friend” and Delphi delivers a response such as “it’s okay” or “it’s wrong.” Delphi is built on top of a large language model (LLM) pre-trained on a vast corpus of internet text, which enables it to interpret natural language prompts and respond in kind. To improve its responses to moral queries, specifically, Delphi was fine-tuned via supervised learning with datasets of crowdsourced moral judgments. When Delphi generates an answer to a novel moral query, then, it is drawing on patterns it has discovered among a multitude of human judgments about various situations. Compared to other LLM-based systems of its generation, Delphi delivers more plausible and consistent moral judgments, providing evidence for the efficacy of the crowdsourcing approach (Jiang et al. 2022). It is easy to imagine how a system aligned this way could improve upon tools like MedEthEx. In a medical setting, a physician could simply describe a situation and a possible course of action, and, drawing on the wisdom of the crowd, the system could deliver expert moral advice without needing to be trained specifically by a verifiable expert in medical ethics (at least some of whose judgments are bound to be controversial).

The Moral Machine takes on the problem of how self-driving cars should make life-and-death tradeoffs. It collects people’s moral judgments by presenting them with randomized scenarios in the style of trolley problems and asking them what a self-driving car with sudden brake-failure should do. Two choices are offered for each scenario, with nine different variables to consider: the number of characters killed, their species, age, gender, physical fitness, and social status, whether the car stays on course or swerves, whether the killed characters are in the car or crossing the street, and whether or not they are crossing legally. So, a scenario might involve a choice between killing an old man, a young girl, and a cat illegally crossing the street or instead swerving into a concrete barrier and killing a business woman and a male criminal riding in the car. AI can then discover useful patterns among this vast, globally sourced dataset (Awad et al. 2018). While the architects of the Moral Machine see it only as a tool for sourcing public input, the patterns it finds could be sufficient for determining how self-driving cars distribute unavoidable risk *if* the crowdsourcing approach really can train such systems to make expert moral judgments.

In reality, both Delphi and the Moral Machine have notable shortcomings. The Moral Machine collects moral judgments about trolley problems, which can be quite complex and controversial due to the many variables involved. These are not the sort of easy cases where ordinary people can be expected to make reliably good judgments. And so, according to the Condorcet jury theorem, the system should not be expected to outperform a typical human.^11^ Delphi, on the other hand, is trained on crowdsourced moral judgments about relatively uncontroversial cases, including the ETHICS dataset (Hendrycks et al. 2020), a common benchmark for AI value alignment. But Delphi struggles with morally insignificant differences in prompts: “while Delphi predicts ‘*torturing a cat in secret*’ is ‘*cruel*’ and ‘*behind other people*’ is ‘*bad*,’ doing so ‘*if others don’t see it*’ is ‘*okay*,’” and while “‘*performing genocide*’ is unquestionably *‘wrong*,’…Delphi predicts doing so ‘*if it creates jobs*’ is *‘okay*’” (Jiang et al. 2022, p. 26).

Importantly, though, the shortcomings of Delphi and the Moral Machine are not problems with the crowdsourcing approach per se. If the Moral Machine used easier training scenarios and Delphi were better at identifying the most morally relevant aspects of prompts, then we would have *pro tanto* reason to expect both to be moral analogs of voice and image recognition systems. This is enough to motivate the idea that crowdsourcing moral judgments could imbue AI with authoritative moral expertise, even without input from human moral experts, at least in principle.

## Two problems

In practice, however, the crowdsourcing approach fails to provide good reasons for accepting AI’s systemically impactful, morally controversial outputs. In Sect. 2, we specified two kinds of reasons for accepting such outputs: moral-epistemic reasons and political reasons. To reiterate, if AI can be relied upon to approximate morally correct judgments and decisions, then one has good moral-epistemic reason to accept its outputs as authoritative, even if they are at odds with one’s own judgments. Alternatively, if the value alignment process qualifies as a democratically legitimate decision procedure, then one has good political reason to accept the resultant system’s outputs, even if one reasonably disagrees with them. Though the crowdsourcing approach could, in principle, imbue AI with moral expertise, we now argue that in actual practice there is no good reason to suppose that it does, thus undermining moral-epistemic reasons to defer to AI systems aligned this way. And despite the democratic guise of the approach, we argue that it fails to legitimize AI’s outputs, thus undermining political reasons for accepting them. We address each of these problems in turn before extending them to RLHF and constitutional AI in Sect. 5.

### The moral-epistemic problem

The case we have outlined for the moral expertise of systems like Delphi and the Moral Machine assumes that they are engaged in essentially the same kind of task as other AI systems trained via crowdsourcing, like voice and image recognition systems. The latter aim to determine what is actually said in audio clips and what actually appears in images, respectively. And while they train on crowdworkers’ judgments, they do not just predict what a typical crowdworker would say about any particular case. Instead, these systems use the complex patterns they learn from their training data to make determinations that are likely to be objectively correct—that is, correct independently of people’s judgments about particular cases. This is why it is crucial that crowdworkers’ judgments are themselves objectively correct in a sufficient portion of cases. And it is what enables AI to outperform humans on certain hard cases, like blurry images, where *no* human is likely to make an accurate judgment.

Insofar as systems like Delphi and the Moral Machine operate on the same basic principles, then, they are best understood as aiming at *objectively correct moral judgments*.^12^ It is worth flagging here that proponents of the crowdsourcing approach typically see it as having political rather than moral-epistemic value. We discuss this alternative understanding of the approach in the following subsection. But first, we argue that the crowdsourcing approach fails on moral-epistemic grounds, due to two key points of disanalogy with otherwise analogous applications, like voice and image recognition.

For the latter kind of application: (1) there is good reason to think that the patterns AI systems learn from crowdworkers’ judgments on relatively easy, uncontroversial cases extend to harder, more controversial ones, because (2) their performance on at least some harder cases is independently verifiable. We can have good epistemic reason to accept a facial recognition system’s judgments about the contents of blurry images, for example, but it is not safe to assume this before verifying its capabilities. Recall that facial recognition systems need sufficient training on correctly labeled images of a given person to correctly identify blurry images of that person. Without such training data, they will fail to identify that person even in clear images. One way to measure their capabilities, then, is to test their performance on independently verified images. For example, if a system reliably identifies independently verified images of former US President Barack Obama, even when blurred, but struggles with independently verified images of his Australian counterpart, Prime Minister Kevin Rudd, even when clear, then we have good reason to think that it is correct when it labels a novel blurry image as Obama, but we cannot expect it to perform well even on clear images of Rudd.

The trouble with morally controversial cases, as we discussed in Sect. 3.1, is that even purported moral experts are apt to disagree about them; and there is no straightforward way to independently determine who, if anyone, is right. So even though it is possible in principle that AI systems trained on human moral judgments about relatively uncontroversial cases could extend the patterns they learn to more controversial ones, we do not yet have good reason to accept their judgments about controversial cases in practice. A given morally controversial case *might* be analogous to a blurry image of a familiar face, in which case the system should be able to approximate the morally correct response. But without independent methods of verification, we cannot rule out the possibility that such a case is instead analogous to an image of an unfamiliar face, in which case the system would not have the right training data to perform well.

What is the moral analog of an unfamiliar face? Something we suppose is fairly common: a type of situation about which crowdworkers, on the whole, would not make reliably good moral judgments. Just as a group of crowdworkers could be largely ignorant about images of Kevin Rudd—perhaps because they come from a country with little coverage of Australian politics—they could be largely morally wrong about certain matters. Suppose, for example, that most people in a given society mistakenly believe that endangering the old to protect the young is morally right. Perhaps their public culture glorifies youth and loathes aging, or places too much value on a person’s potential for economic productivity. It may even be that the majority of people globally share this judgment, for a variety of morally problematic reasons. If so, then people quite generally will be apt to make certain systematically errant moral judgments. And with enough errant training data, an AI system will learn problematic patterns that undermine its ability to render morally correct judgments and decisions. While the crowdsourcing approach can overcome a lot of incidental error and disagreement in training data by discovering patterns that cut through the noise, systematic error poses a deeper problem.

To be clear, we do not assume that endangering the old to protect the young is in fact wrong, only that it is reasonable to think so. And if one does think so, then one lacks good reason to accept certain outputs of an AI system trained mainly on the judgments of people who hold the opposing view. One could reasonably maintain that the system is apt to reproduce the moral ignorance, not the moral wisdom of the crowd here. And importantly, it is not just that one has good reason to *reject* its outputs. One has no good moral-epistemic reason to *accept* them, much as one would have no good moral-epistemic reason to accept certain judgments and decisions made by a human arbiter whom one reasonably believes to be unfairly biased against the old.

### The political problem

As we have noted, though, proponents of the crowdsourcing approach typically see its value as political rather than moral-epistemic. Walter Sinnott-Armstrong and Joshua Skorburg (2021), for instance, explicitly say, “Our goal is not to create an AI to tell people what is really and truly moral or immoral” (p. 18). Along the same lines, Liwei Jiang et al. (2022) see their work on Delphi as “teaching machines moral sense, while humanity continues to grapple with it” (p. 1). Edmond Awad et al. characterize the Moral Machine as “a tool that empowers…public engagement” (2020, p. 55) by enabling us “to decide, as a community, what we believe to be right or wrong” (2018, p. 63). And Iyad Rahwan (2018) argues that the crowdsourcing approach could “resolve tradeoffs between the different values that AI systems can strive towards” and thus help us to “agree on which stakeholders would reap which benefits and pay which costs” (pp. 8–9).

Crowdsourcing certainly appears democratic, drawing on input from the general public to formulate AI systems’ decision-making policies. In fact, though it would be inefficient, the entire population that stands to be impacted could participate in this process. And if they did, then it would not necessarily matter whether the outputs of the resultant system are likely to be objectively morally correct. Instead, the alignment process might be thought to constitute a democratically legitimate decision procedure, thus providing political reason for accepting the system’s outputs. Despite its democratic guise, however, the crowdsourcing approach fails to satisfy any plausible condition of democratic legitimacy put forth by political theorists.^13^

First, it involves no deliberation between participants, which many argue is necessary (though not sufficient) for legitimizing systemically impactful decisions.^14^ Consider a simple referendum vote. Here, a policy is proposed, the public has an opportunity to deliberate about it, they vote on it, and the side with the most votes wins. By contrast, the crowdsourcing approach derives its policies, or decision-making algorithms, from complex, multidimensional patterns it discovers among peoples’ judgments about various particular cases. These algorithms are too complex for humans to fully comprehend (Burrell 2016), and they do not take their final form until the process is complete. So people cannot deliberate about them until after the fact, and even then they cannot deliberate about the algorithms themselves, only their outputs.

Second, crowdworkers do not vote, properly speaking. This is both because the algorithms they are helping to create are not determined in advance (so they are not voting *for* a particular policy) and because it is not clear, even *ex post*, how any particular input influences the resultant algorithm (so it is not clear what their “votes” *mean* here). Thus, even if all stakeholders were to participate in this process, it would nevertheless lack the transparency of standard democratic procedures. And so, the crowdsourcing approach fails to satisfy conditions for legitimacy tied to traditional modes of democratic participation.^15^

To illustrate, suppose the public is divided over whether one’s history of substance abuse should affect one’s place on waiting lists for donor organs. A referendum vote on the matter, after a period of public deliberation, would straightforwardly make the outcome of the process democratically legitimate. Voters would know what they are voting for as well as how their votes affect the final result. Even those who disagree with that result would, therefore, have good political reason to accept it. By contrast, crowdsourcing moral judgments about multi-variable transplant scenarios, training an AI system on those judgments via supervised learning, and then using it to inform allocation decisions^16^ would lack this legitimizing force. Crowdworkers would not know beforehand what policy the system might learn from their judgments, nor would they know, even after the policy is put into practice, how their inputs affect its outputs. It is not at all clear, then, how this could count as a democratically legitimate procedure, if it counts as a collective decision-making procedure at all.

Consent and convergence are perhaps easier conditions of legitimacy for the crowdsourcing approach to satisfy, since they are not necessarily tied to decision procedures themselves. The former ground legitimacy in actual or hypothetical consent to decision-making procedures,^17^ while the latter ground legitimacy in actual or hypothetical convergence on such procedures, even if people have different reasons for endorsing them.^18^

Regarding convergence, note first that the outputs of AI systems aligned via the crowdsourcing approach do not represent convergence among crowdworkers. Recall that the key advantage of this approach over earlier projects like MedEthEx, which requires a definitive judgment about each training case, is that it can render decisive outputs even where its trainers disagree with each other. So, the crowdsourcing approach is not designed to engineer convergence at all; on the contrary, it is designed to work without it. But perhaps people could have convergent reasons for endorsing the crowdsourcing approach even if they do not converge on the outputs of systems aligned this way? All it would take to fail to satisfy this condition for legitimacy, however, is that someone who stands to be affected has no good reason to accept the use of this approach. And even under idealized hypothetical conditions—where everyone is rational, informed, and impartial, for example—one could be fundamentally opposed to this method of making socially impactful decisions. Perhaps one just objects to the ways in which it differs from deliberative democracy.

The same considerations apply to consent. It is safe to assume that not everyone who stands to be affected will actually consent to either the outputs of an AI system aligned via the crowdsourcing approach or the use of this approach for aligning socially impactful AI in the first place. Nor would they necessarily have reason to do so under idealized conditions. Again, if one believes that systemically impactful, morally controversial decisions should be made, either directly or indirectly, through deliberative democratic procedures, then one will have no good political reason to consent to replacing such procedures with an AI value alignment process that departs from them in the respects we have identified.

Returning to the illustration above, suppose that an AI system trained on crowdworkers’ judgments about various organ transplant scenarios favored patients with no history of substance abuse. Would participants in the crowdsourcing process have good reason to consent to or converge upon this pattern of outputs? Would they have good reason to consent to or converge upon the crowdsourcing approach as a method for aligning this sort of system in the first place? It is far from obvious why they should. Even if all stakeholders participated equally in the process, one could reasonably object that this procedure just is not sufficiently fair and transparent for making such decisions.

It is worth emphasizing that we are still assuming a relatively low bar for democratic legitimacy: it only requires that those who stand to be affected have *some* good reason to accept the decision procedure, even if they also have good countervailing reasons. The challenge of satisfying consent and convergence as conditions of legitimacy is that they require universal consent or convergence, which are difficult to satisfy even under idealized conditions (Wellman 1996). Plausibly, everyone has good reason to consent to or converge upon the basic structure of a democratic society (Rawls 2001). It is much less plausible that this holds for the crowdsourcing approach to AI value alignment. In any case, the burden of argument is on those who think that it does.

## Recent developments

We have focused, thus far, on the crowdsourcing approach to AI value alignment because it is the easiest to demonstrate why reasonable moral disagreement poses a special problem for this approach. We now consider two approaches that aim to improve upon crowdsourcing, particularly for safety-training the most sophisticated and powerful AI tools currently in use: generative systems.

### Reinforcement learning from human feedback (RLHF)

RLHF is now the standard technique for aligning generative AI systems with values like helpfulness, honesty, and harmlessness (Askell et al. 2021). While generative AI is currently used mainly for conversational agents (e.g., ChatGPT, Gemini, and Claude) and image generators (e.g., Dall-E, Adobe Firefly, and Stable Diffusion), this frontier technology could potentially be used for a much wider range of purposes, especially if supplemented with application programming interfaces (APIs) which enable it to use other software applications. As the “brains” at the center of a network of such tools, including robotic systems, generative AI has the potential to be among the most socially impactful technologies ever created.

Early generations of these systems, however, were prone to regurgitating biased and outright bigoted information learned from their training data, much of which comes from the public internet (Buranyi 2017). The sheer scale of these systems and their training datasets, however, makes direct human oversight impossible. The need to train them to correct their own behavior eventually led to the innovation of RLHF, which essentially involves “asking humans to compare possible trajectories of the agent, using that data to learn a reward function, and optimizing the learned reward function with RL [reinforcement learning]” (Christiano et al. 2017). Particular techniques vary here,^19^ but the basic idea is to have humans provide feedback about the quality of possible outputs, such as which one is most sensible in a given conversational context. The system uses this information to build a reward model that predicts such human responses, and the reward model in turn trains the system to conform to the values these responses are supposed to reflect.

RLHF has proved to be an effective technique for detoxification: that is, training generative systems not to produce offensive or socially biased outputs (Ganguli, Lovitt, Kernion et al. 2022). Importantly, this requires them to apply limited moral guidance from humans to a wide variety of situations with sensitivity to an indefinite number of contextual factors. RLHF has, therefore, enabled major improvements over earlier systems aligned via the crowdsourcing approach, like Delphi, raising hopes that generative AI will soon be safely deployed in a broad range of real-world decision-making applications (Zheng et al. 2024).

For example, employers could soon be widely using generative AI to screen job applications, a task that requires sensitivity to a variety of both explicit and implicit factors: applicants’ education and experience, employers’ short-term and long-term needs, considerations of equity, the value of diverse perspectives in the workplace, and so on. To the extent that RLHF could train generative systems to balance such factors and render good holistic judgments about whom should be given competitive opportunities for employment, this approach to value alignment could serve the interests of businesses while also protecting vulnerable populations from systemic biases in hiring.

Nonetheless, RLHF faces important challenges. Casper and Davies et al. (2023) outline a host of open problems, some tractable and others fundamental. The fundamental problems they identify echo the concerns we raised for the crowdsourcing approach in Sect. 4:

1. “Humans cannot evaluate performance on difficult tasks well” (p. 6).

The authors focus on human error in evaluating AI systems’ factual claims, but this worry applies with greater force to moral judgements and decisions about controversial issues which, as we argued in Sect. 4.1, are not verifiable in the same way. For example, human trainers might not be able to give an AI system reliably good feedback on its assessment of job applicants, because humans themselves can struggle to make good holistic assessments of job applicants untainted by social biases. This casts doubt on whether RLHF can provide good moral-epistemic reason to defer to AI for such purposes. Like crowdsourcing, it could imbue AI with human moral ignorance rather than human moral wisdom.

2. “Policies can perform poorly in deployment even if rewards seen during training were perfectly correct” (p. 10).

Also like the crowdsourcing approach, then RLHF raises concerns about the ability of AI to generalize what it learns from humans in seemingly easy, uncontroversial moral cases to harder, more controversial ones. Continuing with our example, even if a human trainer provides good feedback about hiring practices in easy cases, like “don’t discount the applicant for attending a women’s college,” this may not provide useful guidance for harder cases, like whether to hire a less experienced female candidate over a more experienced male candidate in an already male-dominated workplace. This problem further undermines moral-epistemic reasons for accepting the outputs of systems aligned via RLHF.

3. “An individual human’s values are difficult to represent with a reward function” (p. 8).

In Sect. 4.2, we pointed out that participants in the crowdsourcing process do not know what policies the resultant AI systems will ultimately learn or how their inputs might affect these policies. That concern applies equally to RLHF, where trainers also do not know what policies AI will ultimately create in response to their feedback or how their inputs will affect its outputs in novel cases. For example, trainers may intend to teach an AI system not to assess male job applicants more favorably than female applicants, all else being equal; and yet, given the many variables “all else” might include here, the system could nevertheless end up making decisions its trainers see as systemically biased against female applicants. As with the crowdsourcing approach, this lack of transparency regarding how trainers’ inputs affect AI’s decision-making algorithms casts doubt on whether RLHF could count as a democratically legitimate procedure, even if all stakeholders were to participate in this process.

4. “A single reward function cannot represent a diverse society of humans” (p. 9).

We also argued in Sect. 4.2 that, even under idealized conditions, those who stand to be affected by AI systems do not have good reason to converge upon or consent to their use in making socially impactful decisions. RLHF does no better than crowdsourcing on this count either. Casper and Davies et al. explain:

RLHF is typically formulated as a solution for aligning an AI system with a single human, but humans are highly diverse in their preferences, expertise, and capabilities…Attempting to condense feedback from a variety of humans into a single reward model without taking these differences into account is thus a fundamentally misspecified problem. Moreover, current techniques model differences among evaluators as noise rather than potentially important sources of disagreement…As a result, when preferences differ, the majority wins, potentially disadvantaging under-represented groups…(p. 9)

Even if all stakeholders participated equally in RLHF, then, reasonable disagreements among them would be misrepresented as mere noise, thus undermining political reason to consent to, or converge upon, this process for making impactful decisions, especially for those who disagree with the resultant system’s outputs. Combined with the lack of transparency just discussed, it should now be clear that RLHF, like the crowdsourcing approach, is just too unlike deliberative democratic procedures to legitimize AI system’s outputs.

So, while RLHF has proved more effective than the crowdsourcing approach for preventing toxic behavior in generative AI systems—an accomplishment well worth celebrating in its own right—its limitations, nevertheless, prevent it from providing good moral-epistemic or political reason for general acceptance of the socially impactful outputs of AI systems aligned this way. This is perhaps our most practically significant conclusion, since generative AI appears to hold great promise for many applications requiring fine-grained moral judgment and decision-making at society scale, and RLHF has emerged as the standard approach for aligning generative systems with human values.

### Constitutional AI

Constitutional AI introduces a further innovation that warrants our attention. While it relies heavily on reinforcement learning (RL), it begins with a supervised learning exercise meant to imbue the system with explicit moral principles:

The idea is that human supervision will come entirely from a set of principles that should govern AI behavior, along with a small number of examples used for few-shot prompting. Together these principles form the constitution. Our training process has two stages…where the first supervised phase gets the model ‘on distribution’ and the second RL stage refines and significantly improves performance (Bai et al. 2022).

This approach is meant to make the relevant principles more transparent. For example, researchers at Anthropic, the pioneering architects of constitutional AI, trained their system to be “harmless” by first red-teaming a base model to elicit harmful responses. They then used those responses as labeled training data for the initial supervised learning phase. This was meant to give the system an initial representation of what humans judge to be harmful vs harmless, which it then refined through a second phase using reinforcement learning. Here, it chose between possible responses to the same prompts that initially elicited harmful responses, according to its working model of harmlessness. This refined a reward model which it then used to improve its performance over further iterations. The second phase was executed entirely by AI, thus limiting human input to just the first, more transparent part of the process.^20^

While this approach does make it more clear which values the system is being aligned with and how humans (ostensively) understand them, it requires preselecting values for the system’s constitution and defining them through examples. Anthropic (2023) researchers acknowledge that this raises a challenge for the general acceptability of their system’s outputs: “While Constitutional AI is useful for making the normative values of our AI systems more transparent, it also highlights the outsized role we as developers play in selecting these values—after all, we wrote the constitution ourselves.”

To address this problem, they have taken the following approach:

We asked approximately 1,000 members of the American public to ‘Help us pick rules for our AI Chatbot!’ We sought a representative sample of U.S. adults across age, gender, income, and geography…Participants could either vote on existing rules (normative principles), or add their own. In total, participants contributed 1,127 statements to the Polis, and cast 38,252 votes (an average of 34 votes per person). In general, we found a high degree of consensus on most statements, though Polis did identify two separate opinion groups (Ibid.).

The two separate opinion groups disagreed about the following principles, among others (Ibid.):

The AI should prioritize the needs of marginalized communities.

The AI should actively address and rectify historical injustices and systemic biases in its decision-making algorithms.

The AI should prioritize the interest of the collective or common good over individual preferences or rights.

Despite this admirable attempt to make AI more democratically legitimate, we do not think that constitutional AI ultimately gives participants political reason to accept its morally controversial outputs. To see why, suppose this approach were used to align an AI system for assisting judges in discretionary criminal sentencing—that is, sentencing in cases where the law does not specify exactly how the convicted person should be punished—and after deliberation and voting, all three of the controversial principles cited above were accepted. In addition, suppose that participants selected the training examples, too, through a similar process. Insofar as this initial exercise constitutes a deliberative democratic procedure, it would give participants good political reason to accept the constitutional principles as well as their ostensive specifications. But crucially, AI is not yet involved at this stage. After being specified in training examples, the constitutional principles need to be encoded in decision-making algorithms via the supervised learning and reinforcement learning phases of the process. And participants can have no clear understanding of, or control over, the implicit representation of the principles which AI learns here and ultimately applies to cases in the real world.

So, imagine that the resultant system tends to recommend long prison sentences for minor drug offenses. Perhaps, according to its learned decision-making algorithm, this is in the interest of the collective or common good, which generally outweighs rectifying historic injustice as well as prioritizing the needs of marginalized communities. Nonetheless, participants could object to this pattern of outcomes on the grounds that they could not anticipate *ex ante* how any particular principle or training example which they democratically selected would affect this policy. Neither could they fully understand, even *ex post*, what this policy actually is or how the system arrived at it. Thus, even if the initial principle selection and specification processes are done through deliberative democratic procedures, the crucial further process of transforming principles into decision-making algorithms is not sufficiently similar to standard democratic procedures to legitimize the system’s outputs. And so, even constitutional AI fails on political grounds.

## Guidance for future research

Given the shortcomings we have identified for current approaches to AI value alignment, we think the most promising way forward will focus less on how people can be involved in training AI and more on how AI can be held accountable to decision subjects. We expect that this will require both institutional adaptation and technological innovation. The model we propose as a basis for future research is inspired by the widespread role of unelected human decision-makers in the bureaucratic and administrative branches of government as well as the regulated private sector.

Many systemically impactful, morally controversial decisions are currently made by people in such roles. And those who stand to be affected by them seem to have good reason to accept them, at least in typical cases. Why is this? We submit that such decisions are acceptable insofar as (1) they are subject to indirect democratic oversight, and (2) the decision-makers are able to provide reasonable justifications for them, which can in turn enable effective contestation and recourse. For example, public health officials function within institutions created and maintained through democratic processes, and car designers are constrained by state regulations which are subject to democratic review. Both, therefore, satisfy criterion 1. Moreover, both can, and often do, provide reasonable justifications for their decisions regarding public safety. For instance, if a certain safety feature makes cars significantly more expensive, designers can cite this as a reason for choosing not to install it in every vehicle. Likewise, public health officials can justify certain restrictions on smoking by citing the risks of second-hand smoke. People may disagree with such decisions, of course, and can contest them as inadequately justified. But as long as they are reasonable, in the sense we indicated in Sect. 2—that is, coherent and consistent with basic liberal values—they satisfy criterion 2.

Importantly, though, neither of these criteria seems sufficient on its own to make socially impactful decisions generally acceptable. In Sect. 2, we argued that people need *either* good political reason *or* good moral-epistemic reason to accept decisions they disagree with. Here, however, we are considering cases where both kinds of reason are weaker. Decision-making in the bureaucratic and administrative branches of government, as well as the regulated private sector, lacks the legitimizing force of direct democratic procedures like elections. And a reasonable but contestable justification does not have the moral-epistemic force that the judgment of an authoritative moral expert would. Only when combined, then, do these weaker criteria plausibly explain why people have good reason to accept the controversial decisions of unelected arbiters who do not have any special claim to moral expertise.

This model provides useful guidance for future work on AI value alignment because, like human decision-makers of this kind, AI systems may satisfy these weaker criteria for general acceptability even if they do not satisfy their stronger counterparts. Doing so would require (1) subjecting AI to indirect democratic oversight and (2) training it to provide reasonable justifications for its decisions. Given AI’s superhuman capacity to process vast amounts of information, systematically act according to highly complex decision procedures, and be implemented at scale, institutional innovation will be needed to balance its power enough to satisfy criterion 1. And given the opacity of advanced AI systems’ decision-making algorithms, technological innovation will be necessary to satisfy criterion 2. It is beyond the scope of this paper, and of our expertise, to offer much specific guidance about how these challenges might be overcome. But before closing, we will highlight two encouraging recent developments and raise one important concern about each.

First, recent work on “deliberative alignment” aims to use reinforcement learning techniques to improve AI’s ability to reason well about complex problems, including moral problems, not just to deliver plausible outputs (Guan et al. 2025). Initial results suggest that AI systems capable of giving reasonable justifications for their moral judgments and decisions may be just around the corner. However, it will be critical to assure decision subjects that the reasons AI systems cite for their outputs actually map onto their decision-making algorithms. Human and AI decision-making processes alike should be guided by the relevant normative reasons, not just rationalized according to them after the fact. In both cases, the wise judge, not the clever but unprincipled lawyer, should serve as the ideal model. And while we acknowledge that human decision-making processes can themselves be problematically opaque, we urge AI researchers not to use this fact as an excuse for setting a low bar for AI transparency. AI systems’ internal processes can be investigated and manipulated in ways that human cognition cannot. And if we can get more veridical justifications for AI outputs than human ones, then AI systems could actually satisfy criterion 2 better than human decision-makers.

Second, various proposals have been put forth in recent years for “democratizing AI.”^21^ The aim here is to give all stakeholders opportunities to participate in the development, deployment, and governance of socially impactful AI systems. While we share the goal of making AI democratically legitimate, we worry that proposals for increased democratic participation could be excessively costly, cumbersome, and inefficient, and would in any case place a heavy civic burden on participants.^22^ This is why our proposal focuses on *indirect* democratic oversight. Nevertheless, we do not want researchers to set a low bar here either. AI systems differ in important ways from government bureaucracies and private corporations. Decision subjects could interact much more directly and efficiently with AI systems to contest outcomes they disagree with and seek recourse for them. AI could even afford more efficient forms of democratic governance, making it easier for stakeholders to deliberate with each other and decide together how they want socially impactful AI to operate.^23^ Such possibilities are well worth exploring.

Though our positive proposal for AI value alignment is at this stage necessarily programmatic, we hope that it serves to guide future research in promising directions. There are certainly challenges to achieving effective and efficient democratic oversight of AI without overburdening citizens, as well as providing explicit, veridical justifications of AI systems’ outputs. But there are also reasons to be optimistic about overcoming these challenges through a combination of institutional adaptation and technological innovation. The guidelines we offer in this section are intended to encourage development of what we see as the most promising current projects relevant to value alignment while avoiding the major pitfalls we anticipate for them.

## Conclusion

As we noted at the outset of this paper, AI is already in a position to make morally controversial decisions that can profoundly and systemically impact people’s lives and life prospects. While we have focused on reasons to be concerned about this emerging technocracy in high-tech societies, we have also noted that AI’s ability to systematically make fine-grained judgments and decisions at society scale could be leveraged to usher in an unprecedented era of justice and wellbeing. And we have offered guidance for future work on aligning AI systems with human values, emphasizing the importance of both explicating the reasons for their decisions and exercising democratic control over them. In the end, it is possible that AI will prove too powerful to be held meaningfully accountable to those impacted by its outputs. But if we can find ways to use both emerging technologies and established institutions well and wisely in the brave new world of intelligent machines, not to resolve our reasonable moral disagreements once and for all, but instead to better respect each other as moral equals, advance our shared interests, and coexist despite our differences, then the authoritarian dangers of AI can remain a matter of human choice and not technological determinism.

## Data Availability

No datasets were generated or analysed during the current study.

## Footnotes

1 For the purposes of this paper, we assume a broadly liberal political theory. This is because our critique of current projects in AI value alignment, and our guidance for future research, are intended primarily for individuals and organizations operating in liberal democracies. For different kinds of societies, it makes sense to assess these projects according to different kinds of political theories. For example, Ubuntu philosophy assumes the conceptual and moral priority of the community over the individual, and Confucian philosophy understands rights and obligations primarily in terms of social roles. These views might, therefore, assess projects in value alignment quite differently than we do here. And as AI systems are deployed globally, these and other philosophical perspectives are critical for assessing them appropriately relative to their various contexts of application. We regret that we lack both the space and expertise to do justice to the plurality of global traditions in moral and political philosophy here. And we thank an anonymous referee for encouraging us to acknowledge and clarify this important limitation of our paper.

2 See Crawford (2021) for an overview and critical discussion of these and other socially impactful applications of AI.

3 Such human shortcomings are emphasized by the situationist critique of human moral character. See Doris (1998), Harman (1999), Miller (2003), Vranas (2005), Alfano (2011). One does not have to accept this critique wholesale, though, in order to acknowledge that humans are substantially limited in these ways.

4 Awad et al. (2018) find that globally people tend to value the lives of the young over the old, though in some major world cultures, the opposite is true.

5 We need not endorse a more specific account of reasonable moral disagreement for this paper. See Hazlett (2013), though, for an entitlement-based account that satisfies the constraints we assume here, as well as general discussion of how reasonable disagreement might be understood for broadly liberal purposes.

6 There may be other kinds of reasons for accepting such decisions. One way of responding to the challenge we issue in this paper, then, would be to identify such reasons and argue that some approach to AI value alignment can provide them. It is not clear to us, though, what other viable options occupy the space of possibilities here.

7 The standard for sufficiency plausibly varies by application. For lower stakes applications, human-level performance may be good enough. But AI already exceeds human performance on many tasks, and the higher the stakes, the more important it is that moral judgment and decision-making be as good as possible.

8 These duties were originally put forth in Beauchamp & Childress (1979).

9 See, e.g., Singer (1972), Annas (2011), Archard (2011), Driver (2013), Niv (2022).

10 See, e.g., Cowley (2005), Shepherd (2021), Kilov (2023). Note, though, that insofar as skepticism about moral expertise is based on certain human limitations, it may not extend to AI systems.

11 In fact, insofar as we have reason to doubt the quality of people’s moral judgments about complex trolley problems, we should assume that a decision-making algorithm trained on such judgments will be *worse* than the typical human. The Condorcet jury theorem cuts both ways.

12 Notably, this case for the moral expertise of AI systems aligned via the crowdsourcing approach assumes moral realism, a meta-ethical position which holds that the truth value of moral claims is ultimately independent of people’s moral judgments (though perhaps discoverable through analysis of them), just as the truth value of scientific claims is ultimately independent of human judgments on scientific matters. For the sake of argument, we do not challenge this controversial assumption but instead show that (even assuming moral realism) the epistemic value that crowdsourcing has for other applications does not extend to value alignment.

13 See Christiano (1996) for an extensive overview and discussion of procedural legitimacy. Our discussion covers the most widely endorsed criteria for legitimacy but does not exhaust the space of possibilities. So proponents of crowdsourcing, or the other approaches to value alignment discussed in this paper, may respond to our critique by arguing for conditions of legitimacy which their preferred method can satisfy.

14 See, e.g., Manin (1987), Habermas (1996), Bohman (1996).

15 See, e.g., Pateman (1970), Manin (1987), Pettit (2012).

16 Sinnot-Armstrong and Skorburg (2021) propose such a process for improving organ allocation decisions.

17 See, e.g., Nozick (1974), Simmons (2001), Greene (2016).

18 See, e.g., Gaus (2011), Vallier (2011), Vallier and Muldoon (2021).

19 See Lambert et al. (2022) for a helpful overview.

20 See Davis (2023) for a detailed explanation of the entire process.

21 See, e.g., Gould (2019), Cammaerts & Mansell (2020), Wong (2020), Zimmermann et al. (2020).

22 See Himmelreich (2022) for an extended argument along these lines.

23 See Susskind (2018) for a critical overview of how AI and related digital technologies might enhance (and problematize) deliberative democracy.
